# Supplementary material for: Fission yeast Rad54 prevents intergenerational buildup of Rad51 aggregates in proliferating cells
Source: Life Sci Alliance. 2025 Aug 18;8(11):e202503252. doi: 10.26508/lsa.202503252 (PMC12360466; doi:10.26508/lsa.202503252)
Supplement: Supplementary file 4 [file LSA-2025-03252_TableS1.docx]

Table S1. Strains used in this study

| Strain | Genotype | Background |
| --- | --- | --- |
| GT414 | *h+ mat1PD17∆::LEU2 ura4-D18 leu1-32* |  |
| GT416 | *h- mat1M smt0 ura4-D18 leu1-32* |  |
| GT422 | *rad54∆::hphMX6* | GT414 |
| GT418 | *rad51∆::kanMX6* | GT414 |
| GT426 | *rad51∆::kanMX6 rad54∆::hphMX6* | GT414 |
| GT479 | *sfr1∆::kanMX6* | GT414 |
| BA269 | *rad57∆::hphMX6* | Argunhan B et al., 2020 |
| GT110 | *rad52∆::natMX6* | GT414 |
| GT476 | *rad54∆::hphMX6 sfr1∆::kanMX6* | GT414 |
| GT473 | *rad54∆::hphMX6 rad57∆::hphMX6* | GT414 |
| GT530 | *rad54∆::hphMX6 rad52∆::natMX6* | GT414 |
| GT295 | *his3∆::rad51-mNeonGreen-kanMX6* | GT414 |
| GT296 | *his3∆::rad51-mNeonGreen-kanMX6* | GT414 |
| GT325 | *his3∆::rad51-mNeonGreen-kanMX6 rad54∆::hphMX6* | GT414 |
| GT440 | *rad52-mCherry2-Tadh1-kanMX6* | GT414 |
| GT506 | *his3∆::rad51-mNeonGreen-kanMX6 rad52-mCherry2-Tadh1-kanMX6* | GT414 |
| GT507 | *his3∆::rad51-mNeonGreen-kanMX6 rad52-mCherry2-Tadh1-kanMX6 rad54∆::hphMX6* | GT414 |
| GT380 | *his3∆::rad51-mNeonGreen-kanMX6 rad52::natMX6* | GT414 |
| GT636 | *rad54-ts-natMX6* | GT414 |
| GT696 | *rad54-ts-natMX6 his3∆::rad51-mNeonGreen-kanMX6* | GT414 |
| GT982 | *ura4+::mCherry-pcn1-natMX* | GT414 |
| GT991 | *his3∆::rad51-mNeonGreen-kanMX6 ura4+::**mCherry-pcn1-natMX* | GT414 |
| MY7270 | *leu1 sad1-mCherry-kanMX* | Nakazawa et al., 2008 |
| GT1137 | *sad1-mCherry-kanMX* | GT414 |
| GT1146 | *his3∆::rad51-mNeonGreen-kanMX6 sad1-mCherry-kanMX* | GT414 |
| GT1053 | *exo1∆::kanMX6* | GT414 |
| BA548 | *his3-D1 arg3-D1 rad54∆::ura4 rqh1∆::natMX6* | Afshar N et al., 2021 |
| GT1050 | *rqh1∆::natMX6* | GT414 |
| GT1175 | *rad54∆::hphMX6* *exo1∆::kanMX6* | GT414 |
| GT1180 | *rad54∆::hphMX6* *rqh1∆::natMX6* | GT414 |
| GT1171 | *rad54∆::hphMX6 exo1∆::kanMX6 rqh1∆::natMX6* | GT414 |
| GT1207 | *his3∆::rad51-mNeonGreen-kanMX6 ura4+::mCherry-pcn1-natMX rad54-ts-natMX6* | GT414 |
| GT1134 | *his3∆::rad51-mNeonGreen-kanMX6 rad54∆::hphMX6 exo1∆::kanMX6 rqh1∆::natMX6* | GT414 |
| GT1254 | *cds1∆::natMX6* | GT414 |
| GT1257 | *chk1∆::natMX6* | GT414 |
| GT1362 | *rad54∆::hphMX6 cds1∆::natMX6* | GT414 |
| GT1272 | *rad54∆::hphMX6 chk1∆::natMX6* | GT414 |
| GT1241 | *cds1-9xPK**-natMX6* | GT414 |
| GT1286 | *cds1-9xPK-natMX6* *rad51∆::kanMX6* | GT414 |
| GT1397 | *cds1-9xPK**-natMX6* *rad54∆::hphMX6* | GT414 |
| GT1295 | *cds1-9xPK-natMX6* *rad51∆::kanMX6* *rad54∆::hphMX6* | GT414 |
| GT1251 | *chk1-9xPK-natMX6* | GT414 |
| GT1300 | *chk1-9xPK-natMX6 rad51∆::kanMX6* | GT414 |
| GT1378 | *chk1-9xPK-natMX6 rad54∆::hphMX6* | GT414 |
| GT1307 | *chk1-9xPK-natMX6 rad51∆::kanMX6 rad54∆::hphMX6* | GT414 |
| GT1414 | *his3∆::rad51-mNeonGreen-kanMX6 rad51∆::kanMX6* | GT414 |
| GT1415 | *his3∆::rad51-mNeonGreen-kanMX6 rad51∆::kanMX6* | GT414 |
| GT396 | *ssb1-mCherry2-Tadh1-kanMX6* | GT416 |
| GT573 | *his3∆::rad51-mNeonGreen-kanMX6* *ssb1-mCherry2-Tadh1-kanMX6* | GT414 |
| GT435 | *his3∆::rad51-mNeonGreen-kanMX6 ssb1-mCherry2-Tadh1-kanMX6 rad54∆::hphMX6* | GT414 |
| GT147 | *rrp1∆::kanMX6* | GT414 |
| GT241 | *fbh1∆::kanMX6* | GT414 |
| GT184 | *rrp1∆::kanMX6* *rad54∆::hphMX6* | GT414 |
| GT951 | *fbh1∆::kanMX6 rad54∆::hphMX6* | GT414 |
| GT1637 | *ade6+::pAde6-pCMV-tetR-P.enotetS-rad54**-**Tadh1-hphMX* | GT414 |
| GT1641 | *ade6+::pAde6-pCMV-tetR-P.enotetS-rrp1-Tadh1-hphMX* | GT414 |
| GT1647 | *ade6+::pAde6-pCMV-tetR-P.enotetS-fbh1-Tadh1-hphMX* | GT414 |
| GT1644 | *ade6+::pAde6-pCMV-tetR-P.enotetS-srs2-Tadh1-hphMX* | GT414 |
| GT1669 | *ade6+::pAde6-pCMV-tetR-P.enotetS-rad54-Tadh1-hphMX rad54∆::hphMX6* | GT414 |
| GT1679 | *ade6+::pAde6-pCMV-tetR-P.enotetS-rrp1-Tadh1-hphMX rad54∆::hphMX6* | GT414 |
| GT1698 | *ade6+::pAde6-pCMV-tetR-P.enotetS-fbh1-Tadh1-hphMX rad54∆::hphMX6* | GT414 |
| GT1687 | *ade6+::pAde6-pCMV-tetR-P.enotetS-srs2-Tadh1-hphMX rad54∆::hphMX6* | GT414 |
| GT1629 | *rev1∆::kanMX6* | GT414 |
| GT1762 | *rev1∆::kanMX6 rad51∆::kanMX6* | GT414 |
| GT1705 | *rev1∆::kanMX6 rad54∆::hphMX6* | GT414 |
| GT1766 | *rev1∆::kanMX6* *rad51∆::kanMX6* *rad54∆::hphMX6* | GT414 |
| GT1626 | *srs2∆::kanMX6* | GT414 |
| GT1515 | *ura4+::Padh1-OsTIR1-F74A cds1-9xPK-natMX6* | GT416 |
| GT651 | *rad54-ts-natMX6* | GT416 |
| GT1771 | *rev3∆::natMX6* | GT416 |
| GT1780 | *rev3∆::natMX6* | GT414 |
| GT1772 | *rev3∆::natMX6 rad51∆::kanMX6* | GT414 |
| GT1775 | *rev3∆::natMX6 rad54∆::hphMX6* | GT414 |
| GT1777 | *rev3∆::natMX6 rad51∆::kanMX6 rad54∆::hphMX6* | GT414 |
