# Supplementary material for: Fission yeast Rad54 prevents intergenerational buildup of Rad51 aggregates in proliferating cells
Source: Life Sci Alliance. 2025 Aug 18;8(11):e202503252. doi: 10.26508/lsa.202503252 (PMC12360466; doi:10.26508/lsa.202503252)
Supplement: Supplementary file 5 [file LSA-2025-03252_TableS2.docx]

Table S2. Primers used in this study

| number | Sequence |
| --- | --- |
| 881 | TTGTTTGAATCCTTATCCCTCTTTTCCTACCCTTTCTTCTATTTCAGTAATCTCTTTTTTAATAATTGCTAATATATTTACGGATCCCCGGGTTAATTAA |
| 882 | GAGAGTAGTTTAAAGACAAATCAAACTAAATGAAAATATGAGGATTTGTGAAGAGAGCAGCGTAATTAAACACGTAAAAAGAATTCGAGCTCGTTTAAAC |
| 772 | TGCTACCTTATCTTTCTTTATTACTTGGTACCTGTCCATGTGCACACAGCTAATATACTGCTTCATATAAGCTAGAAGGGCGGATCCCCGGGTTAATTAA |
| 773 | GATTTGTAATTTCAACAAAATATCAAACAAACGAATTTTCAATTGCAATGGTCAAAACGATTCCCTTTTTAAGAATTTCCGAATTCGAGCTCGTTTAAAC |
| 766 | TGGACAGGTAAAAAAGATCCACGCG |
| 767 | TCTTCCTCATCTCTCACTTGGTAGG |
| 2132 | GTACTACGTTAAAACTGTTACAGAACCAACATCTCGATAACGCACCTGCATTCTTTTGGTGCTTGACGAATGCTTTAAACCGGATCCCCGGGTTAATTAA |
| 2133 | TTGCAACATAAATCGTAATTACTTAAAACCGCACCGTTCATAAAGTAATTAACCCAGTTGGTGATATAATGAGAAAGGTGGAATTCGAGCTCGTTTAAAC |
| 1318 | AACAAATTCTGATCCTCAGTCGGCAATGAGGTCGCGAGAAAACTACGATGCTACGGTGGATAAGAAAGCCAAAAAAGGAATGGTGAGCAAGGGCGAGGAG |
| 1319 | TTAAACAAATCATTAGTCATAAAACAGAAAATACTTGGTAAAAAACAAGTTGCCAATCATCACATTTTGCCTCATTACTTCAGTATAGCGACCAGCATTC |
| 2144 | AACCGATACAGAATGTGATC |
| 2145 | AGGATGCAACGCTGGAAACG |
| 1014 | CTCACTATAGGGCGAATTGGAGCTCCACCGCGGTGGCGGCCGCAATAAGACTGGATTGAACTG |
| 1015 | GAAACGTTCTAATAGGCCAGAAAAGGAAACCCCCGGGCGTATCTCTTTCGATGTGTTTTTGTTAAAG |
| 1016 | CTTTAACAAAAACACATCGAAAGAGATACGCCCGGGGGTTTCCTTTTCTGGCCTATTAGAACGTTTC |
| 1017 | GGGCCCCCCCTCGAGGTCGACGGTATCGATAAGCTGCGGCCGCCCCGAAAAAAGATTAATGAG |
| 1018 | ATAGGGCGAATTGGAGCTCCACCGCGGTGGCGGCCAGACGACTACACGCTATGGAAAAG |
| 1019 | CATAAATCATAAGAAATTCGCCCCGGGTATAACTTGTTAAGCACGAAATTATCAC |
| 1020 | GTGATAATTTCGTGCTTAACAAGTTATACCCGGGGCGAATTTCTTATGATTTATG |
| 1021 | GGGCCCCCCCTCGAGGTCGACGGTATCGATAAGCTCAGTATAGCGACCAGCATTC |
| 1027 | TCTTACTTTAACAAAAACACATCGAAAGAGATACGAGACGACTACACGCTATGGAAAAG |
| 1028 | AATTGAAACGTTCTAATAGGCCAGAAAAGGAAACCCAGTATAGCGACCAGCATTC |
| 1044 | TCATGTTTGCCGCTTGTTGG |
| BA183 | GGCAGATACAGAGGTGG |
| 1562 | TGTAAAACGACGGCCAGTGAGCGCGCGTAATACGACTCACTATAGGGCGAATTGGAGCTCCACCGCGGTGGCGCGGCCGCTATCGCAAATGTTATGAGGC |
| 1563 | GCGCACGTCAAGACTGTCAAGGAGGGTATTCTGGGCCTCCATGTCCTAAAACTGTCACGGGTTCGAATACTAACAAATGC |
| 1564 | GCATTTGTTAGTATTCGAACCCGTGACAGTTTTAGGACATGGAGGCCCAGAATACCCTCCTTGACAGTCTTGACGTGCGC |
| 1565 | CGAGACATCCCAATCTTAATATAGAAAAGCAGTGCAGTATAGCGACCAGCATTCACATACGATTGACGCATGATATTAC |
| 1566 | GTAATATCATGCGTCAATCGTATGTGAATGCTGGTCGCTATACTGCACTGCTTTTCTATATTAAGATTGGGATGTCTCG |
| 1567 | GCGCAATTAACCCTCACTAAAGGGAACAAAAGCTGGGTACCGGGCCCCCCCTCGAGGTCGACGGTATCGATAGCGGCCGCCATATTCTCTCTATCCAACC |
| 2279 | TCACTCATTTGCACGTTTATTTGTGTTTACTGATATACATGGTTAAAGAATTCATCCAGTTTTTCTGTTTTTAAGATACTCGGATCCCCGGGTTAATTAA |
| 2280 | ACTGTCTATTTACAATATTATAAATTTGACGGTCTAAGTATAAAAATTAATTATCATTTAGAATACTAAATATTAATAATGAATTCGAGCTCGTTTAAAC |
| 2281 | GACTGTAAATAATACACATGCCAAAATTTGCAACATATGCTTGTGTTATATTATAATCTTCTTTCCCTTTACACCAAAGACGGATCCCCGGGTTAATTAA |
| 2282 | TTGAAAATAAATTCACAACGAAGTCAAACAACAGGTTTCTTTTACAAACGAACTGGCACGACCTTTCAAAAGATGTGCAAGAATTCGAGCTCGTTTAAAC |
| 2271 | ACACTGTGAGCACACACGAACATCGTACTCCACCATCATCGTCTGAACATGAGGCCACCGAACAGCTCAATTCTTCGAGTCGGATCCCCGGGTTAATTAA |
| 2272 | ACTGTCTATTTACAATATTATAAATTTGACGGTCTAAGTATAAAAATTAATTATCATTTAGAATACTAAATATTAATAATGAATTCGAGCTCGTTTAAAC |
| 2275 | ATTTCATATACTAATCCATGTGAAAGAACGTTGTCAGTTCAATAGGGAAGCCGATTGTTCTTACAGATGTTTCACAAAATCGGATCCCCGGGTTAATTAA |
| 2276 | TTGAAAATAAATTCACAACGAAGTCAAACAACAGGTTTCTTTTACAAACGAACTGGCACGACCTTTCAAAAGATGTGCAAGAATTCGAGCTCGTTTAAAC |
| 1236 | CTATACAGTGATGTCAATTAATCAAATGGACTGGAAGGAAGAGTCTAAAAGATTGATAAATTTCATTGAGTCTGCTCAAATGGTGAGCAAGGGCGAGGAG |
| 1235 | TGCAAAATCATAAATCGTAGTTTTCGCGATTTAAGCCAAAAACTCAATTACTCGACCAGCGAGACGTAAAACACAATGTTCAGTATAGCGACCAGCATTC |
| 877 | CCTCAATTCGATTTTAAAATAATTCTTCCTGGTCTCTTCCTTCTAATCATGTCTCTTTTATAGCCTAGCTCTTAATCATCCGGATCCCCGGGTTAATTAA |
| 878 | ACTACACCACCCCTTTTTTTAACGTTTAGTTTAGTATGCAGTAGAGTATAGTTCATTTGAGTAACGTTAACATTAAATAAGAATTCGAGCTCGTTTAAAC |
| 2673 | TGGCTACATTATTATTTGTATTATTTTCCTTTCAGAAACATATTAATACTTATATTTACTGAAAGTGAGAAGCGCGCTGGCGGATCCCCGGGTTAATTAA |
| 2674 | TCATTATTAAAAAGCATAAACATATCTACTTAAGCGCAAAATCTTTCCCAAAATCCTTATTGAATTTGGAAAACAAAGCAGAATTCGAGCTCGTTTAAAC |
| 2677 | CATACTATGTGAAAACCGACATTTTCTCTCAGCCGTGATAAAAACTATCATATGAAATTTTACTATAAAAAAAGCATTGACGGATCCCCGGGTTAATTAA |
| 2678 | ACTAGCAGGTGATAAGAGAACAAATGCCTGATGTATTTTAATTTCAAGTCCTTCACACCAATATTTGAAGTTATAATGATGAATTCGAGCTCGTTTAAAC |
| 2669 | CTTTGCATTGTTTTCCTATTTCTCACTTTTTTAAAATATACATTTGAAGCTGTCGGTTGAGTGTTTTGTTCTTCTCAATTCGGATCCCCGGGTTAATTAA |
| 2670 | GGTATTTTAAAGAAAACACCGTAGCAAGGTGTTTAGATTGTCTATAAATATAATACGTGATTTTGTACAACATGATATGAGAATTCGAGCTCGTTTAAAC |
| 2690 | ATATATAGAGTGTCCCTATCAGTGATAGAGACTTGTTTCAGTAAGAATCAATTAGTATTCTACAGTAAACATCGGCTAGCATGATTCAGCAACCAACAAC |
| 2691 | TATACACTTATTTTTTTTATAACTTATTTAATAATAAAAATCATAAATCATAAGAAATTCGCTTATTTAGAAGTGGCGCGTTAATGAGATTTGTATTGGA |
| 2692 | ATATATAGAGTGTCCCTATCAGTGATAGAGACTTGTTTCAGTAAGAATCAATTAGTATTCTACAGTAAACATCGGCTAGCATGGATTCATTGTCTGCATA |
| 2693 | TATACACTTATTTTTTTTATAACTTATTTAATAATAAAAATCATAAATCATAAGAAATTCGCTTATTTAGAAGTGGCGCGTCATGAATTAAGCCCAAATA |
| 2696 | ATATATAGAGTGTCCCTATCAGTGATAGAGACTTGTTTCAGTAAGAATCAATTAGTATTCTACAGTAAACATCGGCTAGCATGAGTGCTCAACATTTACA |
| 2697 | AGCCAAGAATAGCAATGGGTATCTCCTTAGGTAATGACACTGATAGCCGAATCGATTGTAGGATTAATTCTTTATTAGTGCGAAAAATAATCGTATGAGG |
| 2698 | CTTCAGCAAGTAATCCCAACAATGTTAGTGGAGCGTTTCGATTTTTTCCTCATACGATTATTTTTCGCACTAATAAAGAATTAATCCTACAATCGATTCG |
| 2699 | TATACACTTATTTTTTTTATAACTTATTTAATAATAAAAATCATAAATCATAAGAAATTCGCTTATTTAGAAGTGGCGCGCTACTGATCATGTACAGCAA |
| 2694 | ATATATAGAGTGTCCCTATCAGTGATAGAGACTTGTTTCAGTAAGAATCAATTAGTATTCTACAGTAAACATCGGCTAGCATGGAAACGAAATCATCATA |
| 2695 | TATACACTTATTTTTTTTATAACTTATTTAATAATAAAAATCATAAATCATAAGAAATTCGCTTATTTAGAAGTGGCGCGCTATAACATTCGTGAAACTC |
| 2677 | CATACTATGTGAAAACCGACATTTTCTCTCAGCCGTGATAAAAACTATCATATGAAATTTTACTATAAAAAAAGCATTGACGGATCCCCGGGTTAATTAA |
| 2678 | ACTAGCAGGTGATAAGAGAACAAATGCCTGATGTATTTTAATTTCAAGTCCTTCACACCAATATTTGAAGTTATAATGATGAATTCGAGCTCGTTTAAAC |
